# Computational polypharmacology comes of age

**DOI:** 10.3389/fphar.2015.00157

**Published:** 2015-07-28

**Authors:** Giulio Rastelli, Luca Pinzi

**Affiliations:** Molecular Modelling and Drug Design Lab, Life Sciences Department, University of Modena and Reggio EmiliaModena, Italy

**Keywords:** polypharmacology, multitarget ligands, drug discovery, drug design, molecular modeling

In the last years, the “one target, one drug” paradigm that has traditionally dominated drug discovery has been deeply challenged by the evidence that small molecules interact simultaneously with multiple targets, a phenomenon known as polypharmacology. Today, polypharmacology is recognized as a new valuable opportunity for drug discovery and development. It is now well established that drug molecules typically bind to several targets, and that their efficacy and safety is mostly dependent on their polypharmacological profile (Jalencas and Mestres, 2012; Peters, 2013; Anighoro et al., 2014). Indeed, one of the most common reasons for terminating a drug discovery program has been promiscuity or lack of selectivity of the developed compounds. This leads to important considerations regarding the polypharmacology inherent in chemical structures and its possible exploitation for drug discovery. First, side effects caused by drug binding to unwanted off-targets (adverse polypharmacology) should be identified as early as possible in the drug discovery pipeline. Second, potential synergistic effects arising from hitting multiple targets (beneficial polypharmacology) should be taken into consideration and thoroughly incorporated in the drug design strategy. Third, polypharmacological approaches have the potential to redirect stalled drug discovery projects and to reposition valuable hits or leads (drug repositioning). Finally, prediction of polypharmacological profiles can be used to uncover new macromolecular targets for already known or new developing drugs (target identification and deconvolution). In all these areas, computational polypharmacology is gaining a foothold in drug discovery, as witnessed by the increasing number of publications reporting theoretical approaches and methods specifically put forward to address these needs.

State-of-the-art computational approaches offer the possibility to predict the activity profile of ligands to a set of targets, thereby anticipating potential selectivity issues or discovering desired multitarget activities early in the iterative design and optimization steps typical of a preclinical drug discovery project. These approaches stem from 2D or 3D shape and chemical similarity, pharmacophore analyses, target and binding site similarity assessment, docking methods, bioinformatics, graph theory and modeling, machine-learning algorithms, and chemogenomics (Figure 1). Broadly, these can be classified into statistical data analysis and bioinformatics, ligand-based, and structure-based approaches, all of which are well documented in the literature (Csermely et al., 2005; Boran and Iyengar, 2010; Bottegoni et al., 2012; Anighoro et al., 2014; Reddy et al., 2014). One should note that ligand-based and structure-based strategies have specific advantages and limitations. Structure-based methods use the information derived from knowledge of the 3D structure of proteins. These methods are applicable to identify ligands for a specific target or set of targets of interest, for example by performing de-novo design or virtual screening of large libraries of small molecules. In addition, they can be used to assess binding site structural similarity and to profile protein-ligand interactions among sets of targets. Their application is obviously limited to proteins with known crystal structure or to homology models derived from highly homologous crystal structure templates. Moreover, structure-based results are influenced by differences in conformations of binding site residues, which are generally difficult to predict. Ligand-based approaches do not require crystal structures of the target proteins but rely on prior knowledge of biologically active ligands, therefore their use is limited to targets for which ligands are known. Worth of note is that in ligand-based methods the derived information is necessarily dependent on the chemical structures of the classes of compounds that have been thus far developed. As a consequence, predicting polypharmacological profiles of ligands that are too dissimilar to already synthesized classes of compounds would be impossible. Overall, ligand-based and structure-based methods appear to be applicable in conjunction to provide more robust results (Anighoro et al., 2015). Such combination offers the possibility to take advantage of the peculiar features and strengths of each approach toward the obtainment of possible candidates for polypharmacology, and appears to be one promising way to go in future investigations. For example, one risk of predicting polypharmacology by using only chemical similarity principles is that inactive compounds can exhibit high similarity with active molecules if they derive from a slight modification of an active compound at some key position crucial for its interaction with the target. In this case such similarity would lead to false positives. Likewise, false negatives can be expected considering that not all of active compounds have been identified for a given target. In these cases, structure-based methods can help overcome these potential pitfalls by estimating the steric and electrostatic complementarity of ligands with the target binding sites. For example, structure-based docking screenings of compounds that passed the desired chemical similarity filters may be independently performed on two or more biological targets of interest, and multi-target hits may be identified from compounds located at the top of all ranked lists. Finally, analysis of drug targets and drug-target associations using a network approach may provide useful information to highlight particularly interesting target combinations or chemical modulators able to perturb the network at specific nodes of disease-specific critical pathways (Csermely et al., 2013). In this context, partial inhibition of a small number of targets can be more efficient than the complete inhibition of a single target, especially for complex and multifactorial diseases (Csermely et al., 2005). This information can be used by ligand-based or structure-based methods to direct the design and screening of new drugs toward the desired set of multiple drug targets.

**Figure 1:**
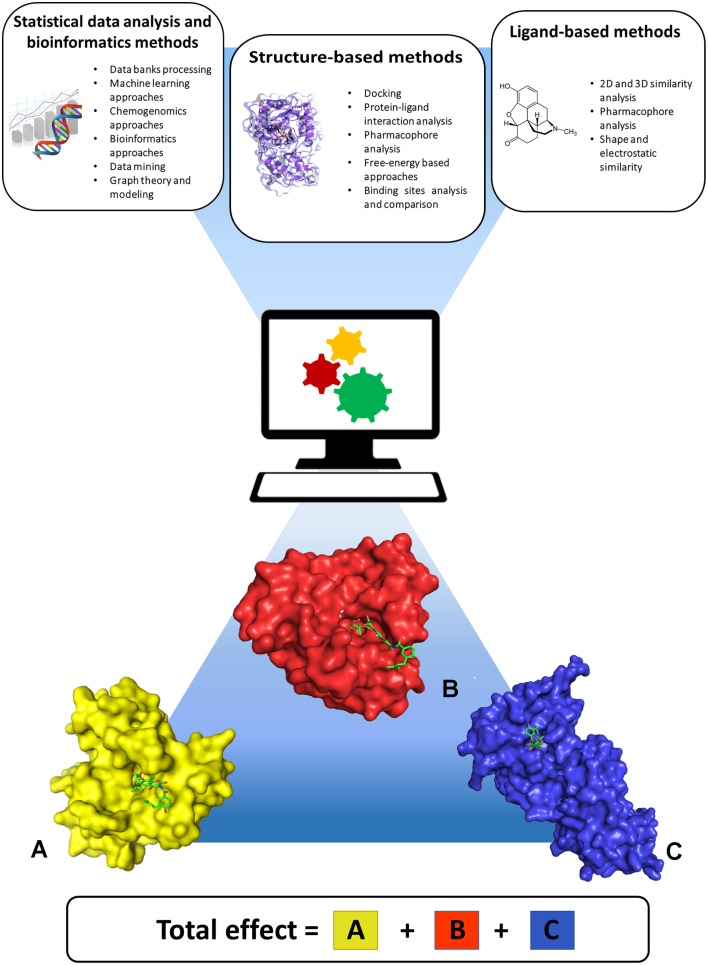
**Computational approaches useful for predicting polypharmacology**. Statistical data analysis and bioinformatics, ligand-based, and structure-based approaches can be applied either singularly or in combination, to take advantage of the peculiar features and strengths of each approach. The lower part of the figure shows three different proteins (A–C) interacting with the same ligand, and highlights that the final pharmacological effect of the ligand is the result of synergistic effects arising from interaction with all targets.

Polypharmacology has been mainly recognized within members of the kinome and GPCRs families (Knight et al., 2010; Jacobson et al., 2014). This is not surprising, considering that binding sites within members of conserved and evolutionarily related targets are generally conserved and thus prone to multitarget inhibition. However, one should note that the recognized specificity of a ligand or a series of ligands depends heavily on how hard on- and off-targets have been investigated, and this is surely the case for kinases and GPCRs, which have been extensively explored. We are far from having the capacity to perform an exhaustive biological profiling of ligands that enter into drug discovery pipelines, but we can expect that the more testing will be performed, the more off-targets and multitarget activities will be seen also for targets genetically and structurally unrelated to the primary intended target. In this respect, constant improvement and implementation of compound and bioactivity data deposited in publicly available databases will provide access to an increasing number of high confidence bioactivity annotations for larger sets of chemicals and therapeutic targets (Hu and Bajorath, 2013). Overall, this information will be very useful to computationally design multitarget ligands. In parallel, improvement in hardware and software performance is making it possible to handle an enormous amount of data, thus enabling the generation and analysis of big data for polypharmacology in a very cost- and time-effective way.

The rational design of molecules interacting with more than one biological target becomes most challenging when these targets are only distantly related or unrelated, i.e., when they belong to different protein families. For example, the selectivity of particularly interesting kinase inhibitors are usually profiled against a large panel of kinases of the kinome, but they are rarely screened against targets of other families due to limited capacity of experimental *in vitro* testing. Considering that local binding site similarities may be more important than global structural similarity to determine polypharmacological activities, especially when ligands are able to interact with key residues of more than one target, this remains a critical point for the development of multi-targeted drugs (Salentin et al., 2014). Therefore, assessing local binding site similarities and comparing protein-ligand interaction profiles, especially for distantly related sets of targets and chemical classes of ligands, will be crucial for predicting polypharmacology (both beneficial and harmful). Significant improvements are also needed on how to select the most relevant set of therapeutically important targets for a given disease, a question that can benefit of the recent progresses of proteomics and clinical molecular investigations on patients and disease states.

Progresses in modeling protein-ligand interactions and in quantitatively predicting free energies of binding of ligands to target proteins will definitely contribute to successful design of molecules with the desired polypharmacological profile. The ongoing advances in docking methods such as improvement of scoring functions and better treatment of receptor flexibility are playing an important role to meet this goal. Importantly, several free-energy based approaches, with different theoretical backgrounds and at different levels of approximation, have been proposed to rescore docking results in order to increase the accuracy of binding affinity predictions (Parenti and Rastelli, 2012). Considering that the affinity of a ligand for a target protein reflects the ΔG of binding, any further improvement in our ability to accurately predict binding free energies will be important to design multi-target drug candidates.

Combining computational design and chemical synthesis of libraries of multi-target ligands provides another means to more effectively obtain bioactive compounds with the desired on- and off-target binding. For example, Reutlinger et al. very recently described the development and application of a computational molecular *de novo* method for designing combinatorial libraries that exhibit an accurately predicted bioactivity profile, obtaining nanomolar multitarget ligands modulating the dopamine D4 and sigma-1 receptors (Reutlinger et al., 2014). In another study, Rodrigues et al. showed that the combination of machine-learning methods with automated chemical synthesis and fast bioassay turnover enabled the generation of small molecules with the desired polypharmacology (Rodrigues et al., 2015). These investigations suggest that a combination of the two approaches may be suitable for rapidly obtaining hits and leads with the desired target engagement.

Finally, a thorough understanding of drug-target network relationships and target-disease associations is key not only to provide more effective and safer drugs, but also to uncover specific target combinations that may provide synergistic effects and/or benefits for mitigating or bypassing drug resistance. In other words, selecting the “right” combination of targets for a specific disease will probably be a major key to success, and this should be given full consideration by focusing computational experiments on target combinations suggested by clinical and/or molecular biology investigations. So far, given the high number of cellular targets and our limited ability to understand their interplay in disease states, most biologically active small molecules are likely to bind several targets and/or to activate or suppress alternate pathways or targets. Network models are providing useful information to analyze the interconnection of pathways and targets relevant to human diseases, and their relation with chemical compound networks (Schadt et al., 2009). However, the more the pathways and mechanisms of disease (especially multifactorial and complex diseases) will be understood at the molecular level, the more the polypharmacological networks can be exploited with computational methods to obtain safer and potent drugs able to modulate the desired on- and off-target activities. The recent successes in *de novo* predicting drug polypharmacology and the raising number of computational strategies and frameworks developed at this purpose testify that computational polypharmacology has come of age and will play an increasingly important role in drug discovery. The combination of different approaches and expertise (experimental and computational) will likely be key to success.

## Conflict of interest statement

The authors declare that the research was conducted in the absence of any commercial or financial relationships that could be construed as a potential conflict of interest.
